# Microglia and neuroinflammation: a pathological perspective

**DOI:** 10.1186/1742-2094-1-14

**Published:** 2004-07-30

**Authors:** Wolfgang J Streit, Robert E Mrak, W Sue T Griffin

**Affiliations:** 1 Department of Neuroscience, University of Florida College of Medicine, P.O. Box 100244, Gainesville, Florida 32610, USA; 2 Department of Pathology, University of Arkansas for Medical Sciences, Little Rock, Arkansas 72205, USA; 3 Department of Geriatrics, University of Arkansas for Medical Sciences and GRECC/CAVHS, Little Rock, Arkansas 72205, USA

## Abstract

Microglia make up the innate immune system of the central nervous system and are key cellular mediators of neuroinflammatory processes. Their role in central nervous system diseases, including infections, is discussed in terms of a participation in both acute and chronic neuroinflammatory responses. Specific reference is made also to their involvement in Alzheimer's disease where microglial cell activation is thought to be critically important in the neurodegenerative process.

## Background

A role for immune responses, involving antigen presentation and immune-response-generating cytokines, in neurodegenerative diseases such as Alzheimer's disease was recognized for a decade before the term neuroinflammation came into widespread use [1,2]. A PubMed search using "neuroinflammation" as the only key word yields some 300 papers, none before 1995 [3]. While some chronic/remitting neurological diseases, such as multiple sclerosis, have long been recognized as inflammatory, the term neuroinflammation has come to denote chronic, CNS-specific, inflammation-like glial responses that do not reproduce the classic characteristics of inflammation in the periphery but that may engender neurodegenerative events; including plaque formation, dystrophic neurite growth, and excessive tau phosphorylation. In this way, neuroinflammation has been implicated in chronic unremitting neurodegenerative diseases such as Alzheimer's disease – diseases that historically have not been thought of as inflammatory diseases. This new understanding has come from rapid advances in the field of microglial and astrocytic neurobiology over the past fifteen to twenty years. These advances have led to the recognition that glia, particularly microglia, respond to tissue insult with a complex array of inflammatory cytokines and actions, and that these actions transcend the historical vision of phagocytosis and structural support that has long been enshrined in the term "reactive gliosis." Microglia are now recognized as the prime components of an intrinsic brain immune system [4], and as such they have become a main focus in cellular neuroimmunology and therefore in neuroinflammation. This is not the inflammation of the adaptive mammalian immune response, with its array of specialized T-cells and the made-to-order antibodies produced through complex gene rearrangements. This is, instead, the innate immune system, upon which adaptive immunity is built [5].

Many researchers now consider this innate immune response in the brain to be a potentially pathogenic factor in a number of CNS diseases that lack the prominent leukocytic infiltrates of adaptive immune responses, but that do have activated microglia and astrocytes, i.e., neuroinflammation.

The idea that neuroinflammation is detrimental implies that glial cell activation precedes and causes neuronal degeneration [2], a sequence of events that appears to be at odds with experimental models of neurodegeneration in which glial cell activation occurs secondary to neuronal damage. What is missing from this simple linear model is the understanding that chronic neurological diseases are just that – chronic, and that this chronicity introduces complex interactions and feedback loops between neurons and glia that render attempts to construct simple, linear cascades of cause and effect inelegant.

In the following, we provide some basic definitions and discussion to more precisely define the idea of neuroinflammation as a CNS tissue response to injury, and the notion of neuroinflammation as a pathogenic factor in neurodegenerative diseases.

### Some basic definitions

Inflammation is a reaction of living tissues to injury [6]. The discipline of pathology makes a fundamental distinction between acute and chronic inflammation. Acute inflammation comprises the immediate and early response to an injurious agent and is basically a defensive response that paves the way for repair of the damaged site. Chronic inflammation results from stimuli that are persistent. In the periphery, inflammation consists of leukocytic infiltrates characterized by polymorphonuclear cells (neutrophils) in acute inflammation and mononuclear cells (macrophages, lymphocytes, plasma cells) in chronic inflammation. In order to validate these principles of general pathology within the context of neuroinflammation, one must obviously consider both acute and chronic neuroinflammation and, therefore, these are addressed separately in the following sections.

### Acute neuroinflammation

Before "neuroinflammation" became a commonly used term, neuroscientists spoke of "reactive gliosis" in describing endogenous CNS tissue responses to injury. Reactive gliosis specifically referred to the accumulation of enlarged glial cells, notably microglia and astrocytes, appearing immediately after CNS injury has occurred. In contrast to glial reactivity, which suggests a largely passive response to injury; glial activation implies a more aggressive role in responding to activating stimuli: activated glial cells release factors that act on and engender responses in target cells analogous to the responses of activated immune cells in the periphery. Activation of immune cells in the periphery leads to leukocyte infiltration of tissues, but this is notably absent in the brain unless there has been destruction or compromise of the blood brain barrier [7,8]. In the presence of such destruction or compromise, peripheral leukocytes do enter the brain producing a scenario similar to that seen in inflammatory responses in the periphery.

In limited, acute reactions to injury, in the absence of blood-brain barrier breakdown, there is the subtler response of the brain's own immune system, composed largely of rapid activation of glial cells. These responses represent the other end of the spectrum of CNS injury, where limited neuronal insults trigger glial cell activation without breakdown of the blood brain barrier and without concomitant leukocytic infiltration. This form of "pure" glial response occurs in neuronal injury caused by either loss of afferents [9] or loss of efferents [10]. Axotomy, for instance, results in neuronal chromatolysis, the classic example of potentially reversible neuronal injury [9]. It is in these situations that microglial and astrocytic responses (like their peripheral counterparts) fulfill their evolutionarily programmed functions of a reparative response to the benefit of the organism as a whole.

Although such specific responses might, in a strict sense, be included in the term "neuroinflammation," neuroinflammation as generally used and understood applies to more chronic, sustained cycles of injury and response, in which the cumulative ill effects of immunological microglial and astrocytic activation contribute to and expand the initial neurodestructive effects, thus maintaining and worsening the disease process through their actions.

### Chronic neuroinflammation

The concept of chronic inflammation (as opposed to acute inflammation) is more relevant in the context of understanding CNS disease (as opposed to CNS injury), as the very term "disease" implies chronicity. Chronic multiple sclerosis is, of course, an unequivocal and long-recognized example of an inflammatory brain disease. Although the underlying cause(s) of multiple sclerosis have not been elucidated, it is probably safe to say that the persistent injurious stimulus that accounts for neuroinflammation in multiple sclerosis is a myelin-related protein that has escaped self-tolerance and become an autoimmunogen. Consistent with the chronic persistence of this autoimmunogen is a persistent accumulation of blood-derived mononuclear leukocytes in the CNS parenchyma, a phenomenon that is similar to what is found in other autoimmune diseases such as rheumatoid arthritis or polymyositis.

Infections are another group of diseases that are classically recognized as inflammatory in nature, with meningeal, perivascular, or even parenchymal infiltrates of peripheral leukocytes. There are, however, exceptions. Rabies is a disease in which the peripheral immune response is slow and inadequate, and in which classic inflammatory changes are less striking than those found in other viral encephalidites. Babes, in 1897 [11], described microglial activation in rabies infection, although he did not recognize the nodules he found as clusters of activated microglia. Similar small collections of activated microglia were subsequently found to occur in a wide variety of viral brain infections.

Today, the most important example of a chronic brain infection is human immunodeficiency virus (HIV). Chronic HIV encephalitis is characterized by the same nodules of activated microglia that Babes described in rabies. HIV enters and persists in the CNS *via *myelomonocytic cells: monocytes, perivascular cells, and microglia [12]. HIV infection is uniquely different from most other infectious diseases affecting the CNS in that the virus targets and disables precisely those cells that are key players in neuroinflammation; microglia in the brain and T lymphocytes in the periphery. It therefore comes as no surprise that prominent T cell infiltrates do not occur in HIV encephalopathy.

Prion diseases represent another chronic infectious CNS disease that is not accompanied by leukocytic infiltrates. Microglial activation, again, appears to be the most prominent inflammatory component of prion diseases [13,14], although there are a few reports describing T cell infiltration as well [15,16]. Prion diseases share interesting parallels to rabies infection in that infected cells are unrecognized by peripheral immune responses. This may explain in part the unusual patterns of neuroinflammation in prion diseases – manifest not only in atypical cellular infiltrates but also in unusual cytokine profiles [17]. Both HIV and prion infections probably produce an altered microglial physiology that is likely to translate into cycles of neurodegeneration, which could be a contributing factor in the development of dementia that occurs in these conditions.

### Chronic microglial neuroinflammation in neurodegenerative diseases

Neurodegenerative diseases – particularly Alzheimer's disease, but also amyotrophic lateral sclerosis, Parkinson's disease, and Huntington's disease – lack the prominent infiltrates of blood-derived mononuclear cells that characterize autoimmune diseases. On the other hand, there is abundant evidence that many substances involved in the promotion of inflammatory processes are present in the CNS of patients with such neurodegenerative diseases. By far the bulk of this body of evidence is related to studies in Alzheimer's disease [18]. What distinguishes Alzheimer's disease from other neurodegenerative diseases is the conspicuous presence of extracellular deposits of amyloid in senile plaques. Senile plaques in Alzheimer brain are present in different stages of maturity, ranging from diffuse to neuritic to dense core, but they all contain the amyloid beta protein (Aβ). Aβ is a peptide that forms insoluble and pathological extracellular aggregates that seem to attract microglial cells, as suggested by the clustering of microglia at sites of Aβ deposition (see [19] for a review). There is evidence from experimental studies in animals to support the idea that microglia can phagocytose and degrade amyloid [20,21], but such phagocytosis is apparently either ineffective or inadequate in Alzheimer's disease. A key question within the current context is: "Does the amyloid in Alzheimer brain by itself represent a persistent injurious stimulus that causes neuronal injury, or are additional factors involved in eliciting this outcome?" Direct injection of Aβ into the brain produces activation of microglia and loss of specific populations of neurons [21]. Furthermore, transgenic mice that overexpress human, mutant β-amyloid precursor protein (βAPP) do develop Aβ deposits with associated evidence of neuritic injury (although they do not develop Alzheimer-type neurofibrillary tangles unless they are also transgenic for human tau protein) [22]. These Aβ deposits, born of transgenic overexpression of mutant human amyloid precursor protein, invariably contain activated microglia [22,23].

β-Amyloid precursor protein βAPP functions as a neuronal acute-phase, injury-response protein. For instance, there is excessive expression of βAPP, accompanied by microglial activation and cytokine expression, after traumatic head injury [24]. With head injury, there is also Aβ deposition, both in experimental animals [25] and in humans – particularly in individuals genetically susceptible for AD (i.e. ApoE ε4-positive) [26]. These observations emphasize the complex interactions that underlie neurodegeneration in Alzheimer's disease.

## Conclusions

Chronic microglial activation is an important component of neurodegenerative diseases, and this chronic neuroinflammatory component likely contributes to neuronal dysfunction, injury, and loss (and hence to disease progression) in these diseases. The recognition of microglia as the brain's intrinsic immune system, and the understanding that chronic activation of this system leads to pathologic sequelae, has led to the modern concept of neuroinflammation. This vision of microglia-driven neuroinflammatory responses, with neuropathological consequences, has extended the older vision of passive glial responses that are inherent in the concept of "reactive gliosis."

## Abbreviations

Aβ: β-amyloid peptide

βAPP: Aβ precursor protein

CNS: central nervous system

HIV: human immunodeficiency virus

MS: multiple sclerosis

## Competing interests

None declared

## Authors' contributions

WJS conceived this review, wrote the initial draft, modified this with the comments of REM and WSTG, and wrote the final draft. REM and WSTG contributed particularly to the sections on infections and on Alzheimer's disease. All authors read and approved the final version.
